# The Programmed Death-1 Pathway Counter-Regulates Inflammation-Induced Osteoclast Activity in Clinical and Experimental Settings

**DOI:** 10.3389/fimmu.2022.773946

**Published:** 2022-03-09

**Authors:** Stinne R. Greisen, Tue W. Kragstrup, Jesper Skovhus Thomsen, Kim Hørslev-Pedersen, Merete Lund Hetland, Kristian Stengaard-Pedersen, Mikkel Østergaard, Lykke Ørnbjerg, Peter Junker, Arlene H. Sharpe, Gordon J. Freeman, Malene Hvid, Søren K. Moestrup, Ellen Margrethe Hauge, Bent Deleuran

**Affiliations:** ^1^ Department of Biomedicine, Aarhus University, Aarhus, Denmark; ^2^ Department of Rheumatology , Aarhus University Hospital, Aarhus, Denmark; ^3^ Danish Hospital for the Rheumatic Diseases , and University of Southern Denmark, Sonderborg, Denmark; ^4^ Copenhagen Center for Arthritis Research, Center for Rheumatology and Spine Diseases, Copenhagen University Hospital Rigshospitalet, Copenhagen, Denmark; ^5^ Department of Clinical Medicine, Faculty of Health and Medical Sciences, University of Copenhagen, Copenhagen, Denmark; ^6^ Department of Rheumatology, Odense University Hospital, Odense, Denmark; ^7^ Department of Immunology, Harvard Medical School, Boston, MA, United States; ^8^ Department of Medical Oncology, Dana-Farber Cancer Institute, Harvard Medical School, Boston, MA, United States; ^9^ Department of Clinical Medicine , Aarhus University, Aarhus, Denmark

**Keywords:** rheumatoid arthritis, PD-1, osteoclast, inflammation, bone homeostasis

## Abstract

**Objective:**

The programmed death-1 (PD-1) pathway is essential for maintaining self-tolerance and plays an important role in autoimmunity, including rheumatoid arthritis (RA). Here, we investigated how membrane-bound and soluble (s)PD-1 influence bone homeostasis during chronic inflammation, exemplified in RA.

**Methods:**

Bone mineral density and bone microstructure were examined in PD-1 and PD-L1 knockout (KO) mice and compared with wild-type (WT) mice. Receptor activator of nuclear factor kappa-B ligand (RANKL) was measured in serum, and the expression examined on activated bone marrow cells. Osteoclast formation was examined in cells from murine spleen and bone marrow and from human synovial fluid cells. sPD-1 was measured in chronic and early (e)RA patients and correlated to markers of disease activity and radiographic scores.

**Results:**

PD-1 and PD-L1 KO mice showed signs of osteoporosis. This was supported by a significantly reduced trabecular bone volume fraction and deteriorated microstructure, as well as increased osteoclast formation and an increased RANKL/OPG ratio. The recombinant form of sPD-1 decreased osteoclast formation *in vitro*, but was closely associated with disease activity markers in eRA patients. Sustained elevated sPD-1 levels indicated ongoing inflammation and were associated with increased radiographic progression.

**Conclusion:**

The PD-1 pathway is closely associated with bone homeostasis, and lacking members of this pathway causes a deteriorated bone structure. The immunological balance in the microenvironment determines how the PD-1 pathway regulates osteoclast formation. In eRA patients, sPD-1 may serve as a biomarker, reflecting residual but clinically silent disease activity and radiographic progression.

## Introduction

The programmed death-1 (PD-1) pathway is essential for maintaining self-tolerance, and if impaired, autoimmune diseases can develop (1, 2). The PD-1 pathway comprises the PD-1 receptor, which is mainly expressed by activated T and B cells, but also by T regulatory cells and exhausted T cells (Tex). PD-1 has two known ligands, PD-L1 and PD-L2. PD-L1 is widely expressed, whereas expression of PD-L2 is restricted to mainly dendritic cells, macrophages, and B cells (3, 4). Activation of the PD-1 pathway decreases inflammation. However, the system is complex and regulates the activation not only of T helper cells but also of regulatory T cells and follicular T helper cells, as well as antibody production (5). In addition, PD-1 is a central mediator in the development of Tex, which are present in persistent inflammatory lesions (6). Since PD-1 expression is increased upon T-cell stimulation, many autoimmune diseases are characterized by upregulation of PD-1 (7, 8). Moreover, studies in mice confirm that impairment of the PD-1 pathway results in increased severity of autoimmune diseases (9–12).

In humans, members of the PD-1 pathway are present in soluble forms readily detected in plasma. The presence of these soluble forms is often associated with increased disease activity, but their functional significance remains to be elucidated in detail (13–15). We previously reported that the PD-1 pathway is associated with bone homeostasis, and in particular, PD-L2 decreases osteoclast activity (16, 17). The association between immune regulatory mechanisms and bone homeostasis has been demonstrated for cytotoxic T-lymphocyte-associated protein 4 (CTLA-4) (18). These observations, and the frequent occurrence of osteoporosis in chronic immune inflammatory diseases, provide further evidence that immune mechanisms are implicated in bone regulatory processes. Pro-inflammatory cytokines like tumor necrosis factor alpha (TNFα) can increase bone destruction (19). This is in part mediated by production of the receptor activator of nuclear factor kappa-B ligand (RANKL), which is the most important inducer of osteoclastogenesis and osteoclast activation, by osteoblasts, activated B cells, T cells, and fibroblasts (20–22).

In rheumatoid arthritis (RA), the joints are the principal target of inflammation, causing pain, swelling, and a gradual destruction of articular cartilage and subchondral bone. This series of events is commonly associated with progressive axial and appendicular bone loss (23, 24). Factors regulating osteoclast activity are present in the inflamed microenvironment in the joint. Synovial fibroblasts express RANKL, while T cells and B cells facilitate inflammation, favoring osteoclast formation and activity. However, as RANKL binds to the decoy receptor osteoprotogerin (OPG) with a higher affinity than to RANKL (25), the ratio between RANKL and OPG determines the availability of RANKL (26).

Despite the availability of a wide range of treatment options in RA, close to 30% of patients experience several treatment failures (27). Moreover, it remains difficult to identify patients at high risk of radiological progression. Further insight into the composition and dynamics of the RA synovial microenvironment, and factors associated with osteoclast activity in particular, is increasingly important to provide tailored therapies.

Therefore, this study aimed to investigate the association between PD-1 and local bone homeostasis and elucidate the role of sPD-1 as a marker of homeostatic and adaptive bone resorption by studies *in vitro*, in murine models, and in patients with rheumatoid arthritis.

## Methods

### Patients and Healthy Controls

All patients in this substudy participated in the OPERA study. In all, 103 patients were included, with an average of 3 months of symptom duration, and randomly allocated to treatment with methotrexate (MTX) + adalimumab (ADA) (anti-TNFα antibody) or MTX + placebo (PLA) (28, 29). Both regimes were administered in combination with corticosteroid injections into swollen joints. After 12 months of treatment, ADA and PLA were discontinued, after which treatment continued with MTX only, adding biologicals in case of disease flare. Plasma samples and clinical and biochemical data were obtained at baseline prior to treatment and at 24 months of follow-up (**Table 1**). Disease scores included C-reactive protein (CRP), IgM rheumatoid factor (RF), and anti-citrullinated protein antibodies (ACPA). Disease Activity Score in 28 joints including CRP (DAS28CRP), Simplified Disease Activity Index (SDAI), Clinical Disease Activity Index (CDAI), global visual analog score (VAS), Health Assessment Questionnaire (HAQ), swollen joint count (SJC), and tender joint count (TJC) were evaluated in both 28 and 40 joints. Radiographs of hands and feet were obtained at baseline and after 24 months and assessed with the modified Sharp–van der Heijde score (total Sharpe score, TSS) (30). From 23 patients with chronic (c)RA, paired plasma and synovial fluid samples were collected at disease flares. All patients had more than 8 years of disease duration, and at sample collection, clinical disease activity parameters were obtained (**Table 1**). Healthy controls (HC, *n* = 38) that were age and gender matched with the OPERA cohort were included.

**Table 1:** Characteristics of early RA (eRA) patients at baseline and after 2 years of treatment and of chronic RA (cRA) patients.

Cohort represented	eRA	eRA	cRA
Disease parameter	Baseline	24 months	>8 years
CRP	30.4 (33.3–37.5)	10.5 (8.1–13.0)^a^	29.8 (10.19–49.5)^b^
DAS28CRP	5.4 (5.2–5.6)	2.3 (2.2–2.5)^a^	5.4 (2.8–8.0)^b^
CDAI	30.2 (27.7–32.7)	3.7 (2.7–4.8)^a^	18.9 (12.9–25.0)^b^
SDAI	32.3 (29.6–34.9)	N/A	N/A
VAS pain	54.4 (49.6–59.2)	19.9 (15.3–24.5)^a^	56.19 (44.7–67.7)^b^
VAS global	58.4 (53.5–63.2)	20.9 (16.2–25.5)^a^	59.95 (48.8–71.1)^b^
VAS doctor	54.0 (49.9–63.2)	6.7 (4.3–9.1)^a^	29.6 (22.4–36.7)^b^
HAQ	1.1 (0.96–1.2)	0.37 (0.26–0.48)^a^	1.045 (0.68–1.4)^b^
SJC28	8.1 (7.0–9.2)	0.15 (0.001–0.3)^a^	2.75 (1.7–3.8)^b^
TJC28	10.7 (9.5–11.9)	0.77 (0.35–1.1)^a^	4.9 (2.0–7.8)^b^
SJC40	11.1 (9.6–12.5)	0.27 (0.0–0.54)^a^	2.6 (1.5–3.6)^b^
TJC40	15.9 (14.2–17.5)	1.5 (0.69–2.2)^a^	4.7 (1.6–7.8)^b^
TSS progression 0–24	N/A	43/103	N/A

CRP, C-reactive protein; IgM-RF, IgM rheumatic factor; ACPA, anti-citrullinated protein antibodies; TSS, total Sharpe score; DAS28CRP, Disease Activity Score in 28 joints including CRP; SDAI, Simplified Disease Activity Index; CDAI, Clinical Disease Activity Index; VAS, visual analog score; HAQ, Health Assessment Questionnaire; SJC28/40, swollen joint count; TJC28/40, tender joint count in both 28 and 40 joints; N/A, not applicable.

a Indicates a significant difference between baseline and 24 months in the eRA cohort.

b Indicates a significant difference between cRA and 24 months eRA.

### Plasma and Cell Preparation

Blood and synovial fluid were collected in EDTA tubes. Plasma and cell-free synovial fluid were collected after centrifugation and kept at −80°C until analysis.

Peripheral blood mononuclear cells (PBMCs) and synovial fluid mononuclear cells (SFMCs) were isolated from blood and synovial fluid by Ficoll gradient centrifugation.

### ELISA on Plasma and Synovial Fluid

Soluble PD-1 levels were measured in plasma and synovial fluid (17) (R&D Systems). The ELISA kit was validated as previously described (31). Soluble PD-1 plasma levels were investigated for correlation with disease scores in both eRA and cRA patients.

### Osteoclast Generation

Osteoclasts were generated from SFMCs, as previously described (32). Cultures were stimulated with 25 ng/ml recombinant human (rh) macrophage colony-stimulating factor (M-CSF) (Peprotech, London, UK) and 50 ng/ml rhRANKL (Abcam, Cambridge, UK). At each media change, stimulations were added, including rhPD-1Fc (1 μg/ml) (R&D Systems, Abingdon, UK), rhPD-L1Fc (1 μg/ml) (R&D Systems, Abingdon, UK), or anti-RANKL (denosumab, 5 μg/ml). All cultures were evaluated after 21 days for tartrate-resistant acid phosphatase (TRAP) activity (B-Bridge International, CA, USA). Matrix metalloproteinase 9 (MMP-9) was measured by ELISA at days 4, 10, and 19 (R&D Systems).

### Osteoclast Staining for Confocal Microscopy

SFMCs were seeded on coverslips and, at day 21, fixed in 4% formaldehyde and blocked in 5% donkey serum (32). Coverslips were stained with primary anti-human antibodies: anti-PD-1 (Abcam, Cambridge, UK), anti-PD-L1 (Beckman Coulter), and matching isotype controls in similar concentration. A secondary antibody was diluted 1:200: donkey anti-mouse 488 (Jackson ImmunoResearch,PA, USA). Coverslips were mounted with DAPI/anti-fade and examined in a confocal microscope (LSM710, Zeiss) equipped with Zen2009 software (Zeiss,Jena, Germany).

### Fibroblast-Like Synoviocyte Cultures

Fibroblast-like synoviocytes (FLS) were generated from cRA SFMCs (33). FLS were evaluated at passages 3–4. Cultures were stimulated with interferon gamma (IFNγ) 10 ng/ml or TNFα 10 ng/ml (both from Peprotech) and subsequently with rhPD-1 (1 μg/ml) or left untreated as a control (34). Flow cytometry staining was performed after 48 h of stimulation, using anti-human antibodies, staining for CD90 FITC (BD, JJ, USA), PD-L1 PeCy7 (BD, JJ, USA), L/D nIR (Thermo Fisher, MA, USA), and RANKL PE (Biolegend). Gating was done on live cells, single cells, and CD90^+^ cells (**Supplementary Figure S1**). The flow cytometry was performed on the LSR Fortessa (BD), and data analysis in FlowJo. MCP-1 was measured by ELISA in the supernatant (Thermo Fisher, MA, USA).

### Mice

Eight-week-old female DBA/1 mice were purchased from Janvier, France. C57BL/6 WT male mice (*n* = 9) were purchased from Taconic, USA. Knockout (KO) mice, PD-1 KO (*n* = 9), and PD-L1 KO (*n* = 10) were all male C57BL/6 mice (*pdcd1^tm1.1Shr^, CD274^tm1Shr^* kindly provided by Prof. Arlene Sharpe), and in total, 28 mice were used in this study. All mice were kept in Scantainers under controlled conditions (21°C–25°C, 30%–60% humidity, and 12-h light/dark cycle).

### Dual Energy X-Ray Absorptiometry

The left femur was pDEXA scanned (Sabre XL, Norland Stratec, NY, USA) at a pixel size of 0.1 × 0.1 mm^2^. Bone mineral content (BMC) and area bone mineral density (aBMD) of the whole femur were determined (35).

### Micro-Computed Tomography

The left distal femoral metaphysis from 8-week-old mice (PD-1 KO: *n* = 7, PD-L1 KO: *n* = 7, WT: *n* = 8) was scanned (Scanco µCT 35, Scanco Medical AG, Wangen-Brüttisellen, Switzerland) with 1,000 projections/180°, an isotropic voxel size of 3.5 µm, an X-ray tube voltage of 55 kV_p_ and current of 145 µA, and an integration time of 800 ms. A 1,000-µm-high volume of interest (VOI) starting 300 µm above the most proximal part of the growth zone containing trabecular bone only was demarcated. Similarly, an 819-µm-high mid-diaphyseal VOI was scanned with 500 projections/180°, an isotropic voxel size of 7 µm, and integration time of 300 ms. A cortical VOI was demarcated with the contour tool of the scanner software thus delineating the periosteal bone surface. The data were Gaussian filtered (*σ* = 0.8, support = 1), threshold filtered (533.8 mg HA/cm^3^), and analyzed (36). 3D visualization was made using Amira 5.6 (FEI Visualization Science Group, MA, USA).

### Serological Bone Biomarkers and Serum Bone Parameters

Blood was collected in centrifugation tubes and serum removed after centrifugation and kept at −80°C until use. RANKL and OPG levels were measured by ELISA (both Abcam).

### Collagen-Induced Arthritis and Disease Evaluation

Nine-week-old DBA/1 mice were immunized at the base of the tail at day 0 with 100 µl type II chick collagen (2 mg/ml) and complete Freund’s adjuvant (CFA) (5 mg/ml). Mice were boosted at day 21 with 100 µl type II chick collagen and incomplete Freund’s adjuvant (all from Chondrex, WA, USA). Mice were scored 3 times per week. Each paw was scored from 0 to 4, making 16 the maximum score per mouse. Scoring was done by experienced personnel, blinded to the group. The mice were sacrificed after 3 weeks of disease, or earlier in accordance with humane endpoints. After 3 weeks, one-third of the mice showed signs of arthritis, and they were injected intraperitoneally (i.p.) with 500 µg/mouse of anti-PD-1 (clone: RMP1-14; IgG2a) (*n* = 7) or anti-PD-L1 (clone: 10F9G2, IgG2b) (*n* = 7), or isotype control for anti-PD-1, IgG2a (BioXcell, NH, USA) (*n* = 6) or for anti-PD-L1, IgG2b (BioXcell, NH, USA) (*n* = 6). The mice were treated once a week, for a total of 3 weeks. A total of 26 mice were included. No mice were excluded. The last score of dead mice was used. Eight mice died in the last week and one mouse in week 2. At the end of the study period, mice were sacrificed, paws were removed, and the femur was cleansed and followed by DXA scan.

### Histological Arthritis Evaluation

Paws were fixed in 4% formalin, decalcified in EDTA, sliced sagittally, and embedded in paraffin. The blocks were cut in 5-µm-thick sections and stained with hematoxylin and eosin. TRAP positivity was determined by enzymatic staining. Arthritis was scored according to Larsson et al. (37). TRAP-positive cell clustered in distinct areas, and the presence of osteoclasts was evaluated by counting the areas with TRAP-positive cells in the paws. TRAP-positive areas were counted in the entire section of the paw/joint. The histological evaluation was performed by an experienced researcher blinded to the group of origin.

### Osteoclast Formation From Mouse Spleen Cells

PD-1 KO, PD-L1 KO, and WT mice were sacrificed at 8 weeks of age, and the spleen was removed. The spleen was homogenized in a tissue grinder. Erythrocytes were lysed in NH_4_Cl. Cells were adjusted to a final concentration of 2 × 10^6^/ml and resuspended in RPMI with the addition of penicillin/streptomycin (5%) and recombinant mouse (rm) M-CSF (50 ng/ml) (R&D Systems, Abingdon, UK) and rmRANKL (25 ng/ml) (R&D Systems) for 7 days. At day 7, cultures were washed and stained for the presence of TRAP-positive cells (B-Bridge International, CA, USA). TRAP-positive multinucleated cells containing three or more nuclei were counted as osteoclasts using microscopic evaluation by a skilled researcher blinded to the group of origin.

### RANKL Expression and Osteoclast Formation From Mouse Bone Marrow Cells

From WT, PD-1 KO, and PD-L1 KO C57BL/6 mice, both femora were collected and cleansed. The bone marrow cells were harvested by centrifugation at 1,600*g* for 2 min. Cells were resuspended in RPMI media and filtered, pooled, and kept at −150°C.

Bone marrow cells were cultured in the presence of phorbol myristate acetate (PMA) (Peprotech) for 48 h, and the expression of RANKL was investigated by flow cytometry using anti-mouse antibodies, staining for RANKL AF647 (BD), CD45 FITC (BD), CD11b PeCy7 (BD), and L/D nIR (Thermo Fisher). (**Supplementary Figure S2**). The flow cytometry was performed on the Quanteon (Agilent, CA, USA), and data analysis in FlowJo.

Bone marrow cells were also cultured for osteoclast differentiation. Cells were cultured in DMEM with penicillin/streptomycin (5%), fetal calf serum (10%), and Glutamax (5%) and stimulated with PMA and TNFα (10 ng/ml). After 8 days in culture, cells were stained for TRAP positivity and evaluated as previously described.

### Study Approval

Human clinical studies were conducted in accordance with the Helsinki declaration. All patients provided written informed consent to participate in the study. Studies were approved by the Danish Data Protection Agency, the Danish Medical Agency, and the Regional Ethics Committee (2012–1329–2). Plasma from HCs was obtained from an established cooperation with the Danish blood bank, and HC cannot be traced or identified. DBA/1 animal studies and studies on bone marrow cells from C57BL/6J were performed in Aarhus, Denmark, and approved by the Danish Animal Experiments Inspectorate, protocol nos.: 2014-15-0201-00001 and 2004-15-0201-00001. Other studies involving C57BL/6J WT and KO mice were performed in Boston, USA, and approved by the Harvard Medical Standing Committee on Animal Welfare, protocol number 1214.

### Statistics

Statistical analyses were performed in Stata 13 and GraphPad Prism. Normally distributed data were analyzed using parametric statistics; otherwise, they were analyzed using non-parametric test: Mann–Whitney *U*-test or Wilcoxon matched pairs signed-rank test. Correlations were investigated by regression analysis or Spearman’s correlation. All regression analyses were adjusted for confounders, including age, gender, smoking, and days since diagnosis.

Other data sets were analyzed using parametric statistics when applicable and non-parametric when data did not meet the criteria for the normal distribution; *p*-values <0.05 were considered statistically significant. Data are presented as (mean ± SD) or median (IQR).

## Results

### The PD-1 Pathway Is Important for Optimal Trabecular Bone Density and Structure *In Vivo*

**Figure 1:**
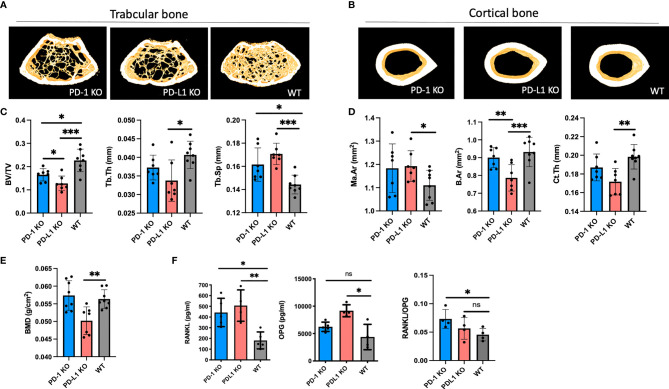
BMD decreases in programmed death-1 (PD-1) knockout (KO) mice. **(A)** Representative images of trabecular bone from microCT scanning of PD-1 KO, PD-L1 KO, and WT mice (*n* = 7–8). **(B)** Representative images of cortical bone from microCT scanning of PD-1 KO, PD-L1 KO, and WT mice (*n* = 7–8). **(C)** Trabecular bone parameters in 8-week-old PD-1 KO, PD-L1 KO, and WT mice (*n* = 7–8). **(D)** Cortical bone parameters in 8-week-old PD-1 KO, PD-L1 KO, and WT mice (*n* = 7–8). **(E)** Bone mineral density of 8-week-old PD-1 KO, PD-L1 KO, and WT mice (*n* = 7–8). **(F)** Levels of receptor activator of nuclear factor kappa-B ligand (RANKL) and osteoprotogerin (OPG) in serum from WT and KO mice at 8 weeks of age. Levels of RANKL are significantly increased in KO mice, whereas levels of OPG are only increased in PD-L1 KO mice. The ratio between RANKL/OPG is significantly decreased in PD-1 KO mice (*n* = 4–5). All mice are male. * indicates *p* < 0.05, ** indicates *p* < 0.01, and *** indicates *p* < 0.001; ns, not significant. Ma.Ar, marrow area; B.Ar, bone area; Ct.Th, trabecular thickness; BV/TV, bone/tissue volume; Tb.Sp, trabecular spacing; Tb.Th, trabecular thickness; BMD, bone mass density. Data are presented as mean with SD.

In trabecular bone, the bone density was significantly reduced and the bone microstructure was significantly deteriorated at 8 weeks of age in both PD-1 KO and PD-L1 KO mice (**Figure 1A**). Thus, the trabecular bone volume fraction (BV/TV; PD-1 KO: 16% ± 2.7%, PD-L1 KO: 13% ± 3.1%, WT: 23% ± 1.7%, *p* < 0.05) and trabecular spacing (Tb.Sp) were significantly decreased in both PD-1 KO and PD-L1 KO mice compared with those in WT. Trabecular thickness was only decreased in PD-L1 KO mice (**Figures 1A, C** and **Supplementary Figure S3**).

The diaphyseal cortical bone was mainly affected in PD-L1 KO mice, with a decreased bone area (B.Ar; PD-L1 KO: 0.79 ± 0.07 mm^2^, WT: 0.93 ± 0.08 mm^2^, *p* < 0.05), as well as a decreased cortical thickness (Ct.Th) and an increased marrow area (Ma.Ar) (**Figures 1B, D** and **Supplementary Figure S1**). Additionally, the whole femoral area bone density was assessed by DEXA. The areal bone mineral density was significantly lower in PD-L1 KO mice compared with that in WT mice, while it did not differ between the PD-1 KO mice and WT mice (**Figure 1E**). In 16-week-old mice, the bone density in PD-L1 KO did not change, whereas it decreased in PD-1 KO mice (**Supplementary Figures S3A, B**).

### The RANKL Production Is Increased in PD-1 KO and PD-L1 KO Mice

We next investigated whether the RANKL/RANK/OPG pathway was affected in the KO mice. RANKL and OPG were measured in serum from WT and KO mice. RANKL levels were significantly increased in both PD-1 KO (442.7 ± 132 pg/ml) and PD-L1 KO (507.2 ± 147 pg/ml), compared with those in WT (181.9 ± 79 pg/ml). The decoy receptor for RANKL, OPG, was only increased in PD-L1 KO mice, as also evidenced by the RANKL/OPG ratio being increased only in the PD-1 KO mice (**Figure 1F**).

### Increased Osteoclast Formation *In Vitro* From PD-1 and PD-L1 KO Bone Marrow Cells

**Figure 2:**
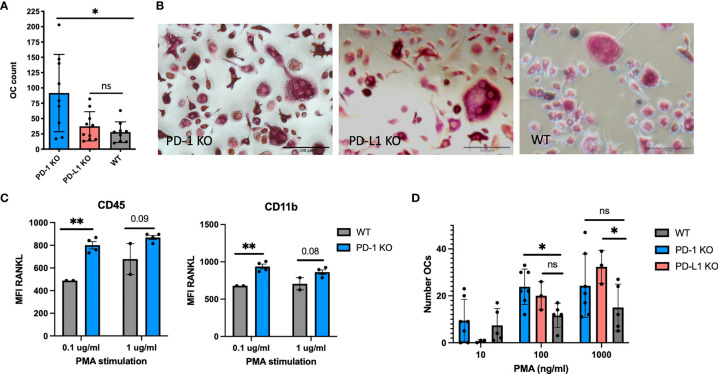
Osteoclast formation from mouse hematopoietic cells. **(A)** Mouse spleen cells from WT and KO mice were stimulated with RANKL and macrophage colony-stimulating factor (M-CSF) for 7 days and tartrate-resistant acid phosphatase (TRAP) stained. Large multinucleated cells were counted. More osteoclasts form from PD-1 KO spleen cells (*n* = 9), PD-L1 (*n* = 10), and WT (*n* = 9). **(B)** Representative microscopy images of osteoclast cultures from WT, PD-1, and PD-L1 KO mice as described in **(A)**. **(C)** Pooled bone marrow cells from WT and PD-1 KO mice stimulated with the indicated concentration of PMA and investigated by flow cytometry. RANKL MFI increases upon stimulation of KO cells (WT: *n* = 2, KO: *n* = 4). **(D)** Generation of osteoclasts from WT, PD-1 KO, and PD-L1 KO bone marrow cells by PMA stimulation. TRAP-positive cells are counted on day 7. All wells are counted twice and the average count is presented in the figure. Data are from three individual experiments (PD-1 KO: *n* = 7, PD-L1 KO: *n* = 3, WT: *n* = 5). * indicates *p* < 0.05 and ** indicates *p* < 0.01; ns, not significant. MFI, median fluorescence intensity. Data are presented as mean with SD. All countings are repeated twice.

We proceeded to study osteoclast formation from spleen cells following stimulation with RANKL and M-CSF. From PD-1 KO cells, significantly more osteoclasts developed (91.7 ± 63), than from WT cells (27.8 ± 17), consistent with the structural bone changes. However, as opposed to what was observed on the bones, osteoclast formation did not differ between WT and PD-L1 KO spleen cells (**Figures 2A, B** **)**.

Bone marrow cells were harvested from both KO models, and to mimic a non-specific inflammatory condition, the cells were cultured in the presence of PMA and evaluated for RANKL expression and osteoclast formation. Cells did not express RANKL prior to stimulation and no difference was observed in the percentage of RANKL between WT and KO bone marrow cells prior to stimulation (data not shown). The RANKL MFI was significantly higher on CD11b PD-1 KO (937 ± 64) cells compared with that on WT (679 ± 0) after stimulation with 0.1 μg/ml PMA. The same was observed for CD45^+^ cells (**Figure 2C**). No change was observed for PD-L1 KO cells (data not shown). We also evaluated the expression of PD-L1 on PD-1 KO and WT cells; however, no change in PD-L1 expression was observed (**Supplementary Figure S3C**). Evaluation of osteoclast formation after stimulation with PMA revealed that the number of osteoclasts developed from KO cells increased with increasing concentrations of PMA (**Figure 2D**).

### Collagen-Induced Arthritis Is not Aggravated by Anti-PD-1 Antibody Treatment

We investigated whether disease activity and bone mineral density were affected by treatment with antibodies blocking the PD-1 pathway in DBA/1J mice with established collagen-induced arthritis (CIA).

**Figure 3:**
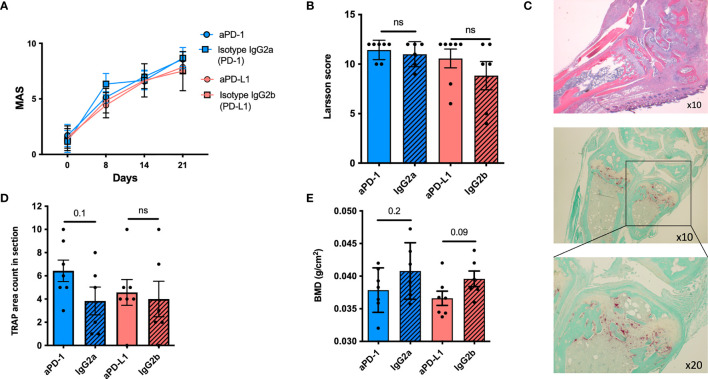
The PD-1 pathway in mice with collagen-induced arthritis. **(A)** Mean arthritis score (MAS) in DBA mice with collagen-induced arthritis (CIA) treated with anti-PD-1 (*n* = 7) (blue) or anti-PD-L1 (*n* = 7) (red) or the corresponding isotype controls (*n* = 6). No difference was observed. **(B)** Larsen score in paws after histological staining. No difference was observed. **(C)** Representative H&E images of a mice paw from a mouse treated with anti-PD-1 (top panel) and stained for the presence of osteoclasts using enzymatic staining for TRAP (red cells) (bottom panel); ×10 and ×20 refer to lens magnification. **(D)** The presence of osteoclasts was evaluated by counting the areas with TRAP-positive infiltrating cells. Mice treated with anti-PD-1 tended to have more TRAP-positive areas compared with the isotype controls. **(E)** The femur was removed for DXA scanning and bone mineral density (BMD) was measured. Though a tendency, no significant differences were observed. All data are presented as mean with SD. ns, not significant.

No difference was observed between the treatment groups as assessed by the disease score MAS. However, all mice presented with severe disease: all MAS >8 (**Figure 3A**). In accordance with the clinical score, no difference was observed between the groups, when evaluating inflammation in the paws, using the Larsen scoring system (**Figure 3B**). Since the PD-1 pathway potentially modulates osteoclast formation, paws were stained for TRAP to identify osteoclasts (**Figure 3C**). Mice treated with neutralizing anti-PD-1 antibodies had a numerical higher number of TRAP-positive cells in relation to their joints and bones (**Figure 3D**) when compared with isotype-treated mice (6.4 ± 2.4 vs. 3.8 ± 2.9, *p* = 0.1). Finally, the bone mineral density was evaluated. Potentially explained by the brief treatment period, no significant difference was observed; however, antibody-treated mice tended to have a lower BMD than isotype-treated mice (**Figure 3E**).

Several studies suggest that the soluble form of PD-1 plays a role in inflammation (13–15). Based on this and our above findings, we next studied whether the soluble form of PD-1 and PD-L1 would influence osteoclast formation using human cells.

### Soluble PD-1 Decreases Osteoclast Formation *In Vitro*

**Figure 4:**
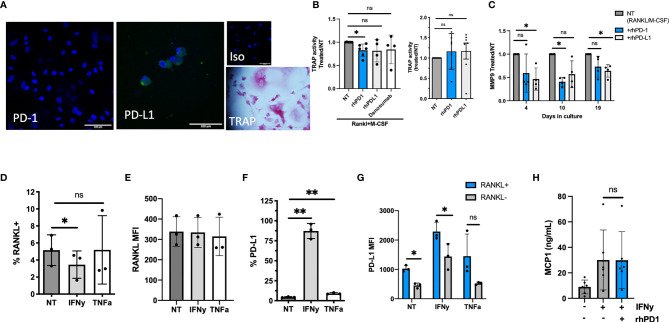
The function of soluble (s)PD-1 in RA synovial fluid mononuclear cell (SFMC) cultures. **(A)** Confocal staining images of an SFMC osteoclast culture at day 21. First image showing PD-1 staining, with no positive cells. Second image showing PD-L1 expressing cells, then isotype control. Final image showing light microscopy and TRAP staining of an osteoclast. Blue: DAPI, green: PD-1 or PD-L1. **(B)** Evaluation of the effect of sPD-1 in human osteoclast cultures. Under the influence of RANKL and M-CSF, rhPD-1 decreased TRAP activity at day 21 (*n* = 4–7). The recombinant proteins did not influence TRAP activity in non-stimulated cultures (right panel) (NT, non-treated). Data are presented as treated/NT. **(C)** MMP-9 in SFMC osteoclast cultures treated with RANKL and M-CSF, and the recombinant proteins at days 4, 10, and 19. MMP-9 in the supernatant decreased significantly in the presence of rhPD-1 and rhPD-L1. Data presented as the ratio between RANKL/M-CSF treated (=NT) and cultures with added recombinant proteins. Data represent the average of two individual setups (*n* = 4). **(D)** Fibroblasts from the arthritic joint express RANKL, and the expression is largely independent on the stimulation (*n* = 3). **(E)** Median fluorescence intensity (MFI) of RANKL in fibroblast-like synoviocytes (FLS). No difference in the MFI between stimulated and NT FLS is observed (*n* = 3). **(F)** Surface expression of PD-L1 on RA FLS. Upon IFNγ stimulation, levels reach close to 100%. The levels also increased upon TNFα stimulation (*n* = 3). **(G)** Evaluating PD-L1 on RANKL+/RANKL− cells. Data expressed as MFI. Higher MFI of PD-L1 was observed on RANKL-positive cells (*n* = 3). **(H)** FLS culture left non-stimulated or stimulated with IFNγ and then with the addition of rhPD-1. MCP-1 levels increase upon stimulation with IFNγ but are unaffected by the addition of rhPD-1 (*n* = 5). All data are presented as mean with SD. *represents *p* < 0.05 and ** represents *p* < 0.01; ns, not significant.

We first examined whether sPD-1 or sPD-L1 would affect osteoclast formation *in vitro*. We used our previously validated model, culturing RA SFMCs to osteoclasts (32). After 21 days of cell culturing, we detected expression of PD-L1, but not PD-1 among the cells in the culture (**Figure 4A**). We identified osteoclasts as TRAP-positive cells (**Figure 4A**). Next, we investigated whether the addition of rhPD-1 or rhPD-L1 to the culture could influence osteoclast formation, assessed by TRAP activity in the supernatant. The addition of rhPD-1 to SFMC cell cultures stimulated with rhRANKL and rhM-CSF decreased TRAP activity after 21 days. Though not significant, a similar observation was made when adding either rhPD-L1 or denosumab (a RANKL inhibitor that mimics endogenous OPG). A high concentration of RANKL could possibly explain the less clear effect seen with denosumab (**Figure 4B**, left). Adding rhPD-1 and rhPD-L1 did not affect the spontaneous formation of osteoclasts (**Figure 4B**, right panel).

As the SFMC culture is a mixture of several different cell types, we next considered whether the PD-1 pathway could affect other pathways in relation to joint homeostasis. We evaluated MMP-9 as MMPs are closely involved in the breakdown of extracellular matrix in many tissues, including bone, where MMP-9 is expressed on osteoclasts (38). Adding rhPD-1 and rhPD-L1 decreased the level of MMP-9 in these cell cultures (**Figure 4C**).

RANKL expressed by FLS is a potent inducer of osteoclast activity in RA, and we confirmed that RA FLS express RANKL [non-treated (NT): 5.1% ± 1.8%] (**Figures 4D, E** **)**. Upon stimulation, PD-L1 was also expressed by FLS (NT: 4.4% ± 0.6% vs. IFNγ: 87% ± 9% and TNFα: 9.4% ± 1%) (**Figure 4F**). In particular, RANKL-positive FLS expressed PD-L1 (**Figure 4G**). We next considered if engaging PD-L1 by rhPD-1 would affect FLS activity. MCP-1 was used as a broad marker of fibroblast activation and increased significantly upon IFNγ activation, but it was unaffected by the addition of rhPD-1 (**Figure 4H**).

### Soluble PD-1 Is Increased in Patients With Early RA and Correlates With Disease Activity and Progression

**Figure 5:**
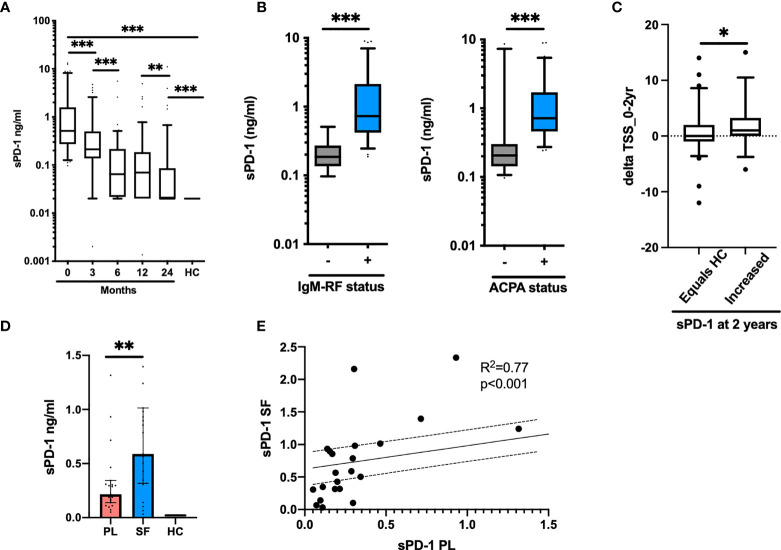
Soluble PD-1 in early and chronic RA. **(A)** Levels of sPD-1 in early RA patients (*n* = 103). Levels decrease significantly from baseline (0 months) until after 2 years of treatment. **(B)** sPD-1 levels at baseline in patients positive for IgM-RF (left panel) and ACPA (right panel). Levels are significantly higher in patient with autoantibodies. **(C)** Delta TSS from baseline to 2 years of treatment in patients with a sPD-1 as HC (equals HC) and an increased sPD-1 (increased) after 2 years of treatment. **(D)** Levels of sPD-1 in chronic RA patients. Levels are significantly increased compared with HC and levels in the synovial fluid are higher than in plasma. **(E)** sPD-1 levels in plasma and synovial fluid correlate, following a linear regression pattern. All data are presented as median with 5–95 range. * represents *p* < 0.05, ** represents *p* < 0.01, and *** represents *p* < 0.001.

Finally, we studied the levels of sPD-1 in plasma from eRA patients. In eRA patients, the levels of sPD-1 did not differ between the two treatment arms, so they were considered as one. Soluble PD-1 levels were significantly increased in eRA patients as compared with those in HC [sPD-1: 0.51 ng/ml (0.27–1.6), HC: 0.02 ng/ml (0–0), *p* < 0.001] and decreased after treatment initiation. The levels remained higher than in HC, even after 2 years of treatment, where 35/103 patients still presented with increased levels of sPD-1 (**Figure 5A**). sPD-1 levels in both IgM-RF and ACPA-positive patients were significantly higher than those in IgM-RF and ACPA-negative patients (**Figure 5B**). Baseline levels of sPD-1 were associated with CRP, DAS28CRP, SDAI, CDAI, SJC 28 + 40, and TJC 28 + 40, but only in the seropositive patients (**Table 2**).

**Table 2:** Correlation between plasma sPD-1 and disease activity parameters at baseline in early RA patients (*n* = 103).

Disease parameter	Correlation sPD-1
CRP	4.3 (0.12–8.5), *p* = 0.04
DAS28CRP	0.13 (0.009–0.25), *p* = 0.035
SDAI	2.0 (0.42–3.6), *p* = 0.014
CDAI	1.9 (0.43–3.5), *p* = 0.013
SJC28	0.75 (0.088–1.4), *p* = 0.027
SJC40	1.2 (0.3–2.1), *p* = 0.01
TJC28	0.84 (0.077–1.6), *p* = 0.031
TJC40	1.2 (0.15–2.3), *p* = 0.026

Data are analyzed by a multiple regression analysis with the correlation coefficient (confidence interval) and subsequent p-value. p < 0.05 is considered significant.

CRP, C-reactive protein; IgM-RF, IgM rheumatic factor; ACPA, anti-citrullinated protein antibodies; TSS, total Sharpe score; DAS28CRP, Disease Activity Score in 28 joints including CRP; SDAI, Simplified Disease Activity Index; CDAI, Clinical Disease Activity Index; VAS, visual analogue score; HAQ, Health Assessment Questionnaire; SJC28/40, swollen joint count; TJC28/40, tender joint count in both 28 and 40 joints.

Based on the entire cohort, no associations were detected between sPD-1 levels at baseline and delta TSS after 2 years of treatment. However, in patients with persistently elevated sPD-1 levels at 2 years of treatment, delta TSS (0–24 months) was significantly higher in patients with increased sPD-1 versus those in whom sPD-1 had reached the levels of HC (1.9 ± 3.8 vs. 0.64 ± 3.9) (**Figure 5C**). Among patients with persistently increased sPD-1, 61% progressed radiographically (defined as delta TSS > 0) as compared with 32% of patients with sPD-1.

### Soluble PD-1 Is Increased in the Inflamed RA Joint

In plasma from cRA patients with disease flare, sPD-1 levels were significantly increased compared with those from HC, and the levels were even higher in the synovial fluid (**Figure 5D**). In both plasma and synovial fluid, sPD-1 levels correlated to VAS global (both Spearman *p* ^2^ = 0.6 and *p* < 0.01). We did not observe correlations with other markers of disease activity. The levels in plasma and synovial fluid correlated (**Figure 5E**). Soluble RANKL was undetectable in both plasma and synovial fluid (data not shown).

## Discussion

In this report, based on experimental and clinical studies, we provide evidence that silencing the PD-1 receptor or its ligand PD-L1 leads to severe signs of osteoporosis, with loss of trabecular and cortical bone. Moreover, we show that in the local microenvironment the soluble forms of PD-1 and PD-L1 can counteract osteoclast formation, demonstrated *in vitro*. However, high systemic levels of sPD-1 in RA patients are associated with more inflammation and radiographic progression. The observations in KO mice are similar to what we have previously demonstrated for PD-L2 KO mice (16). By contrast, Nagahama et al. have demonstrated PD-1 deficiency to cause mild osteopetrosis (39). However, in their study, the transmembrane-encoding exon for PD-1 was deleted in the KO mice, whereas in our studies, both the extracellular and the transmembrane exons were deleted from the PD-1 KO mice. Differences in age and gender might also influence the discrepancies, as bone homeostasis differs between female and male mice (40). Wang et al. showed that inhibiting PD-1 signaling in a mouse model of bone cancer decreased bone destruction (41). This suggest that the PD-1 pathway promotion on cancer growth is superior to its role in bone homeostasis.

Bone homeostasis is closely regulated through balanced degradation and formation. Increased inflammation drives degradation, clinically manifested by increased occurrence of osteoporosis in chronic inflammatory conditions (42). We demonstrated an increase in osteoclast formation in spleen cells from PD-1 KO mice, but not in spleen cells from PD-L1 KO mice. Spleen cells represent a more diverse immune environment and bone marrow cells represent a more direct line of osteoclast formation, which is why we investigated osteoclast formation at both sites. Though the osteoclasts formed from the spleen cells were small in size, they were TRAP positive and were multinucleated. The increased RANKL/OPG ratio in serum and increased RANKL cellular expression on activated bone marrow cells could partly explain the increased osteoclast formation in these mice (43, 44). In line with these data, CTLA-4, another co-inhibitory receptor, is also known to influence osteoclast bone homeostasis by decreasing osteoclast formation (18).

We next investigated if we could influence bone homeostasis in arthritic mice by blocking the PD-1 pathway after disease induction. All mice developed severe disease, and we did not observe any differences between the mice with regard to inflammation. However, we did observe a trend toward increased numbers of osteoclasts in the subchondral bone from mice treated with PD-1 antibodies. In line with this, we also observed a trend toward a decreased BMD in the same mice. These data are supported by case reports from cancer patients treated with anti-PD-1 antibodies, showing an increased rate of severe bone fractures (45). Since we did not include dynamic bone histomorphometry in our analysis, we cannot determine conclusively whether the decreased BMD is caused by increased bone resorption or reduced bone formation. However, data from both the µCT analysis and the *in-vitro* findings support the notion that osteoclasts play a role when the PD-1 pathway is not functional.

In RA and other inflammatory conditions, the presence of sPD-1 is suggested to be associated with disease activity. These *in-vitro* and animal experimental studies clearly support the notion that the PD-1 pathway serves osteoprotective purposes. However, the soluble form of PD-1 could serve as a marker of inflammation in human RA similar to acute phase proteins (46, 47). Soluble PD-1 levels were high prior to treatment, decreased as inflammation subsided, and were associated with other biochemical markers of inflammation. Continuous antigen presentation sustains inflammation, which supports the association between sPD-1 and autoantibody seropositivity in eRA. Some patients have persistently high sPD-1 levels, probably reflecting persistent subclinical disease activity as observed in imaging studies, which may explain why radiographic progression is particularly prominent in this subset of patients (48).

Our observation of an association between high baseline sPD-1 and radiographic progression in seropositive RA patients is intriguing and calls for future studies on the biology of sPD-1. This is further supported by a recent finding suggesting that sPD-1 may antagonize the function of PD-1 and aggravate arthritis in a CIA mouse model (15). On the other hand, administration of sPD-L1 improved disease activity in a CIA mouse model—supporting increased signaling through the PD-1 pathway (49). As we established that the addition of sPD-1 and sPD-L1 to *in-vitro* osteoclast cultures decreased osteoclast formation, the complex interplay between PD-1, PD-L1, and the soluble forms is a balanced outcome, dependent on multiple factors in the microenvironment. The composition of the cellular infiltrate in the joint, which recently has been proposed for subclassification and management of early inflammatory arthritis, may also play a role in determining the local effects of the interplay between the soluble and the membrane-bound forms (50). The PD-1 pathway is complex, which is also highlighted by studies of the cellular expression of PD-1, which can be associated with both immune activity and immune control. A reduced cellular expression of PD-1 is observed in juvenile arthritis, suggesting that some autoimmune diseases might be characterized by dysfunctional co-inhibitory pathways causing increased inflammation (51).

Although we have examined eRA patients with symptoms for less than 3 months, the results will be afflicted by some degree of heterogeneity. This is further the case when proceeding to our *in-vitro* cultures, using SFMC from chronic arthritis patients. In all our *in-vitro* cultures, we use rhPD-1 as a substitute for sPD-1. The negative control for the recombinant protein was left blank, as no matching control could be obtained. Our study is strengthened by the inclusion of a well-established animal RA model, *in-vitro* studies, and well-characterized patients with RA at early and advanced stages.

In conclusion, we demonstrate a significant role for the PD-1 pathway in maintaining a healthy bone structure and regulating osteoclastogenesis. We suggest that sPD-1 and sPD-L1 could function to decrease osteoclast formation in a local environment; however, the systemic functional role of sPD-1 warrants further investigation. Finally, we show that sPD-1 is a promising candidate marker for both clinically active and silent but progressive disease in eRA.

## Data Availability Statement

The raw data supporting the conclusions of this article will be made available by the authors, without undue reservation.

## Ethics Statement

The studies involving human participants were reviewed and approved by the Danish Ethics Committee. The patients/participants provided their written informed consent to participate in this study. The animal study was reviewed and approved by the Danish Animal Experiments Inspectorate.

## Author Contributions

All authors listed have made a substantial, direct, and intellectual contribution to the work and approved it for publication.

## Funding

Funding from Lundbeck Foundation (Grant number: R287-2018-1094), Gigtforeningen, and Aarhus University was granted to SG and BD. The µCT scanner was donated by the VELUX Foundation.

## Conflict of Interest

GF and AS have patents/pending royalties on the PD-1 pathway from Roche, Merck, Bristol-Myers-Squibb, EMD-Serono, Boehringer-Ingelheim, AstraZeneca, Dako, and Novartis. GF has equity in Nextpoint, Triursus, iTeos, and Xios. GF has served on advisory boards for Roche, Bristol-Myers-Squibb, Xios, Origimed, iTeos, and Nextpoint. AS has served on advisory boards for Novartis, Surface Oncology, Elstar, SQZ Biotechnologies, Adaptimmune, Elpiscience, and Monopteros. AS has received research funding from Novartis, Roche, UCB, Ipsen, Merck, and Quark.

The remaining authors declare that the research was conducted in the absence of any commercial or financial relationships that could be construed as a potential conflict of interest.

## Publisher’s Note

All claims expressed in this article are solely those of the authors and do not necessarily represent those of their affiliated organizations, or those of the publisher, the editors and the reviewers. Any product that may be evaluated in this article, or claim that may be made by its manufacturer, is not guaranteed or endorsed by the publisher.

## Supplementary Material

Supplementary Figure 1 Gating strategy for the fibroblast panel. **(A)** Gating was done on forward side scatter gating away debris. Then gating on single cells. **(B)** subsequently on live cells and on CD90+ cells. Further gating was done on FMO’s. **(C)** from left: RANKL FMO, RANKL expression in non-treated fibroblasts (NT), RANKL expression in IFNy treated fibroblasts. **(D)** from left: PD-L1 FMO, PD-L1 expression in non-treated fibroblasts (NT), PD-L1 expression in IFNy treated fibroblasts.

Supplementary Figure 2 Gating strategy for mouse bone marrow cells. **(A)** Gating was done on all cells, then on single cells and live cells. Subsequent gating was done on the subpopulation CD45+ and CD11b+.

Supplementary Figure 3 Bone parameters for PD-1 KO, PD-L1 KO and WT mice. **(A)** cortical and trabecular bone parameters. SMI, Structual modal index; TbN, trabecular number pr mm; pMOI, polar Moment of Inertia; TA, trabecular area. **(B)** Dexa scanning of the femur. BMD, bone mineral density. All *n* = 7–8. All male mice and 8 weeks of age unless others specified. **(C)** PD-L1 expression in CD11b positive bonemarrow cells from PD-1 KO and WT mice.
