# Supplementary figures and images for: The Programmed Death-1 Pathway Counter-Regulates Inflammation-Induced Osteoclast Activity in Clinical and Experimental Settings
Source: Front Immunol. 2022 Mar 9;13:773946. doi: 10.3389/fimmu.2022.773946 (PMC8959817; doi:10.3389/fimmu.2022.773946)

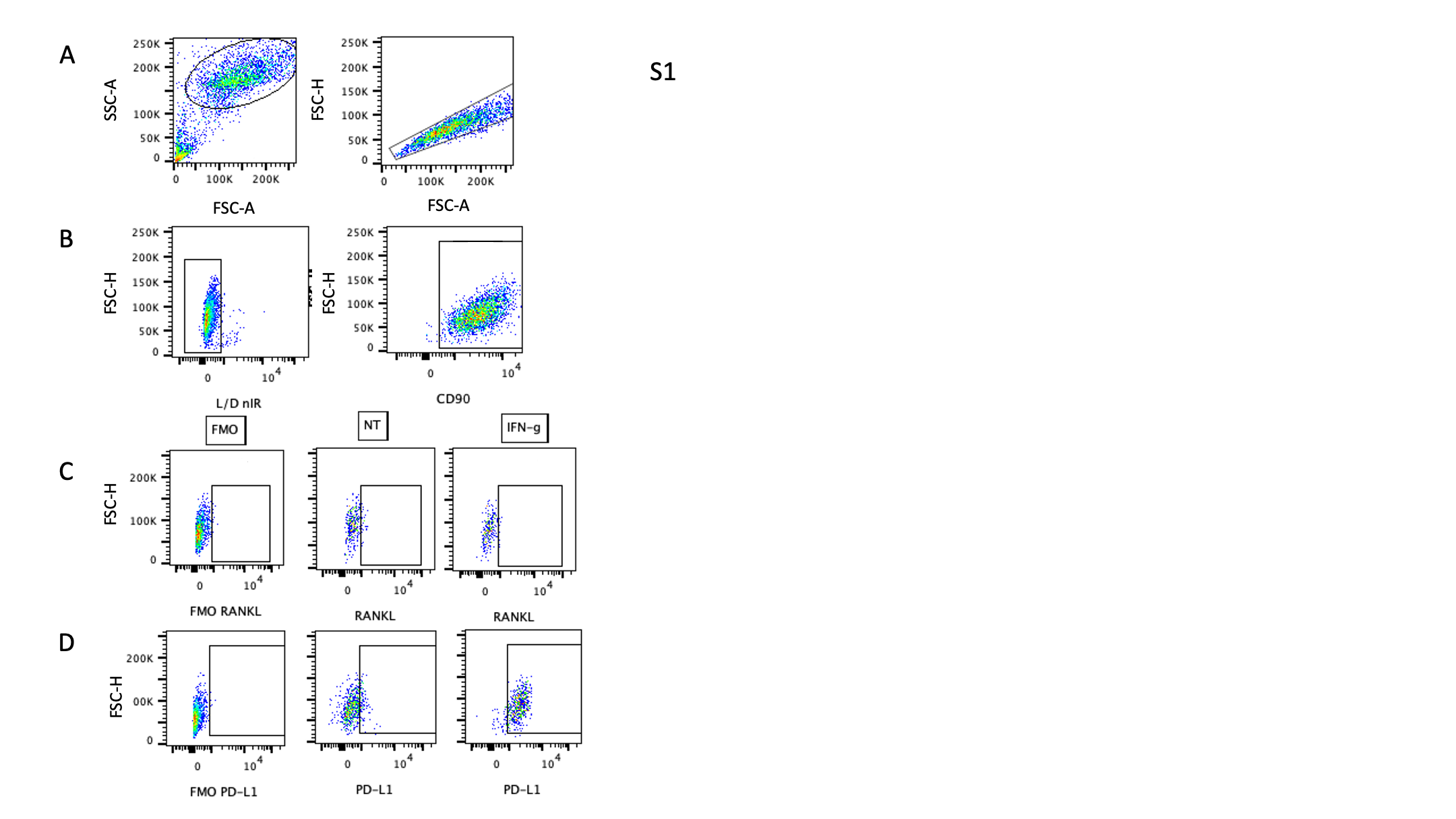

Supplement: Supplementary Figure 1 — Gating strategy for the fibroblast panel. (A) Gating was done on forward side scatter gating away debris. Then gating on single cells. (B) subsequently on live cells and on CD90+ cells. Further gating was done on FMO’s. (C) from left: RANKL FMO, RANKL expression in non-treated fibroblasts (NT), RANKL expression in IFNy treated fibroblasts. (D) from left: PD-L1 FMO, PD-L1 expression in non-treated fibroblasts (NT), PD-L1 expression in IFNy treated fibroblasts. [file Image_1.tiff]

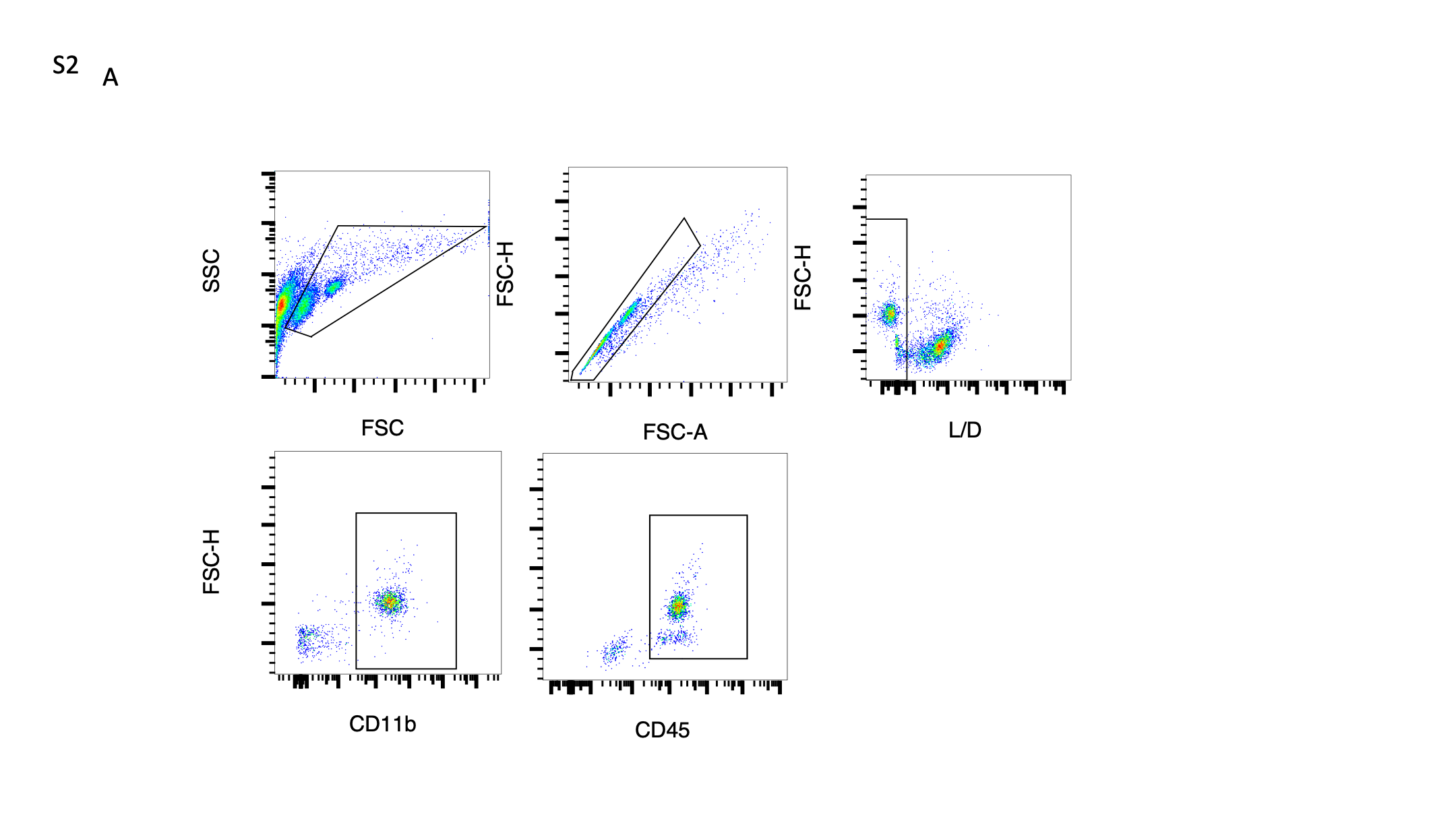

Supplement: Supplementary Figure 2 — Gating strategy for mouse bone marrow cells. (A) Gating was done on all cells, then on single cells and live cells. Subsequent gating was done on the subpopulation CD45+ and CD11b+. [file Image_2.tiff]

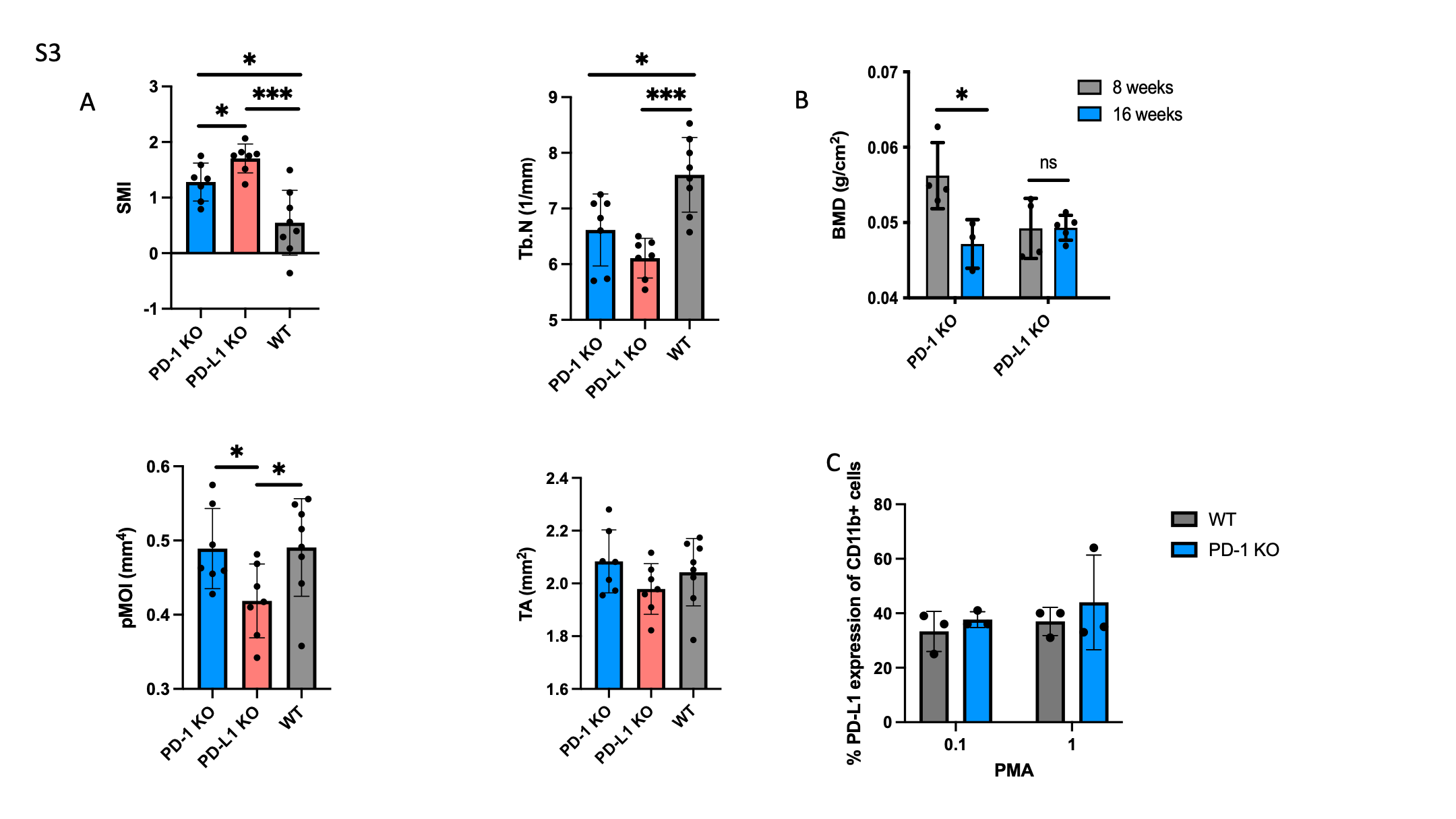

Supplement: Supplementary Figure 3 — Bone parameters for PD-1 KO, PD-L1 KO and WT mice. (A) cortical and trabecular bone parameters. SMI, Structual modal index; TbN, trabecular number pr mm; pMOI, polar Moment of Inertia; TA, trabecular area. (B) Dexa scanning of the femur. BMD, bone mineral density. All n = 7–8. All male mice and 8 weeks of age unless others specified. (C) PD-L1 expression in CD11b positive bonemarrow cells from PD-1 KO and WT mice. [file Image_3.tiff]
